# The proteomic analysis of breast cell line exosomes reveals disease patterns and potential biomarkers

**DOI:** 10.1038/s41598-020-70393-4

**Published:** 2020-08-11

**Authors:** Yousef Risha, Zoran Minic, Shahrokh M. Ghobadloo, Maxim V. Berezovski

**Affiliations:** 1 Department of Chemistry and Biomolecular Sciences, University of Ottawa, Ottawa, Canada; 2 John L. Holmes Mass Spectrometry Facility, Faculty of Science, University of Ottawa, Ottawa, Canada; 3 Cellular Imaging and Cytometry Facility, Faculty of Science, University of Ottawa, Ottawa, Canada

**Keywords:** Proteomics, Breast cancer

## Abstract

Cancer cells release small extracellular vesicles, exosomes, that have been shown to contribute to various aspects of cancer development and progression. Differential analysis of exosomal proteomes from cancerous and non-tumorigenic breast cell lines can provide valuable information related to breast cancer progression and metastasis. Moreover, such a comparison can be explored to find potentially new protein biomarkers for early disease detection. In this study, exosomal proteomes of MDA-MB-231, a metastatic breast cancer cell line, and MCF-10A, a non-cancerous epithelial breast cell line, were identified by nano-liquid chromatography coupled to tandem mass spectrometry. We also tested three exosomes isolation methods (ExoQuick, Ultracentrifugation (UC), and Ultrafiltration–Ultracentrifugation) and detergents (*n*-dodecyl β-d-maltoside, Triton X-100, and Digitonin) for solubilization of exosomal proteins and enhanced detection by mass spectrometry. A total of 1,107 exosomal proteins were identified in both cell lines, 726 of which were unique to the MDA-MB-231 breast cancer cell line. Among them, 87 proteins were predicted to be relevant to breast cancer and 16 proteins to cancer metastasis. Three exosomal membrane/surface proteins, glucose transporter 1 (GLUT-1), glypican 1 (GPC-1), and disintegrin and metalloproteinase domain-containing protein 10 (ADAM10), were identified as potential breast cancer biomarkers and validated with Western blotting and high-resolution flow cytometry. We demonstrated that exosomes are a rich source of breast cancer-related proteins and surface biomarkers that may be used for disease diagnosis and prognosis.

## Introduction

Small extracellular vesicles (sEVs) are membrane-enclosed vesicles secreted by many cell types^1^. Exosomes, a subset of sEVs, have gained significant attention due to their ability to facilitate intercellular communication between distant and nearby cells. These 50–120 nm particles are found in many biological fluids (such as milk, urine, blood, and saliva)^2,3^ and house proteins, metabolites, and nucleic acids such as DNA and RNA^4–6^. Their unique biogenesis, through the endosomal pathway, enables their selective enrichment with functionally relevant proteins capable of influencing several diseases, including cancer^7^.

In breast cancer (BC), exosomes have been shown to drive different stages of carcinogenesis, such as metastasis. By transferring their content to other cell lines, exosomes from the highly invasive and migratory breast cancer cell line Hs578Ts(i)8 increased the proliferation, migration, and invasion capabilities of less aggressive BC cell line variants^8^. It has been reported that exosomes promote invasive behavior in the tumor microenvironment. Fibroblast derived exosomes are able to activate the Wnt/PCP (planar cell polarity) signaling pathways boosting BC cell motility^9^. Furthermore, BC tumor-derived exosomes can attenuate the immune response. BC-derived exosomes induce myeloid immune cells into myeloid-derived suppressor cells suppressing anti-tumor immune response^10^. Exosomes from the highly metastatic BC cell line EO771 suppressed the proliferation of T-cells. These vesicles transformed T-cells into “exhausted T-cell” with immune inhibitory receptors, causing cancer cells to evade the immune system in their microenvironment^11^. Furthermore, exosomes enable the spread of chemotherapy resistance. Docetaxel drug-sensitive cells developed resistance to the drug after being subjected to exosomes derived from non-responsive, resistant, cancer cells. This is hypothesized to be the result of the exosomal transfer of P-glycoprotein, which disposes of many foreign substances out of cells^12^.

Due to their selective enrichment and ease of accessibility from biofluids, exosomes are an attractive source of biomarkers for disease detection and prognosis^13^. Saliva and urine exosomal proteins have already been proposed as biomarkers for different cancers, including non-small lung carcinoma, prostate, and gastric cancers^14–16^. Furthermore, plasma exosomes are a source of early disease biomarkers for Alzheimer’s and Parkinson’s neurological diseases ^17,18^.

Modern mass spectrometry (MS)-based proteomic methodologies have been extensively used for disease-specific biomarker discovery studies, offering high sensitivity capable of identifying low abundant proteins over wide dynamic ranges^19^. This technology has been applied extensively over the years to identify cancer biomarkers in clinical drug development and disease prognosis^20–22^. Therefore, using MS‐based proteomics approaches, we attempted to analyze BC exosomal proteins to discover new BC biomarkers. Extracted exosomal proteins from the triple-negative metastatic cancer cell line MDA-MB-231 (MDA) and the control immortalized epithelial breast tissue cell line MCF-10A (MCF) were analyzed by nano-liquid chromatography-tandem mass spectrometry (nLC-MS/MS). Among the unique MDA proteins, analyzed eight proteins were identified as potential biomarkers, and three candidate proteins were validated.

## Methods

### Cell culturing

MDA epithelial breast cancer cells (ATCC HTB-26) and MCF non-tumorigenic epithelial breast cell line (ATCC CRL-10317) were used in this study. MDA cells were maintained in high-glucose Dulbecco’s Modified Eagle’s medium (DMEM, Gibco) and supplemented with 10% fetal bovine serum (FBS, Corning). MCF cells were grown in DMEM/Nutrient Mixture F-12 (DMEM/F12) supplemented with 5% horse serum (HS), 20 ng/mL epidermal growth factor (EGF), 0.5 mg/mL hydrocortisone, 100 ng/mL cholera toxin and 10 µg/mL insulin. Cell culture media, for both cell lines, were supplemented with 100 units/mL penicillin and 0.1 mg/mL streptomycin (Pen Strep, Gibco). Cells were incubated at 37 °C with 5% CO_2_.

### Exosome isolation

Cells were grown in complete media; when cells reached 40–50% confluency, they were rinsed with Dulbecco modified phosphate-buffered saline (PBS) and incubated with exosome free media for 48 h. Exosome free media was prepared by ultracentrifugation of FBS or HS at 100,000*g* for 17 h. The vesicle enriched media was then collected and processed for the isolation of exosomes. Isolation optimization tests were carried out on MDA-derived exosomes.

#### Differential ultracentrifugation (UC)

**Figure 1:**
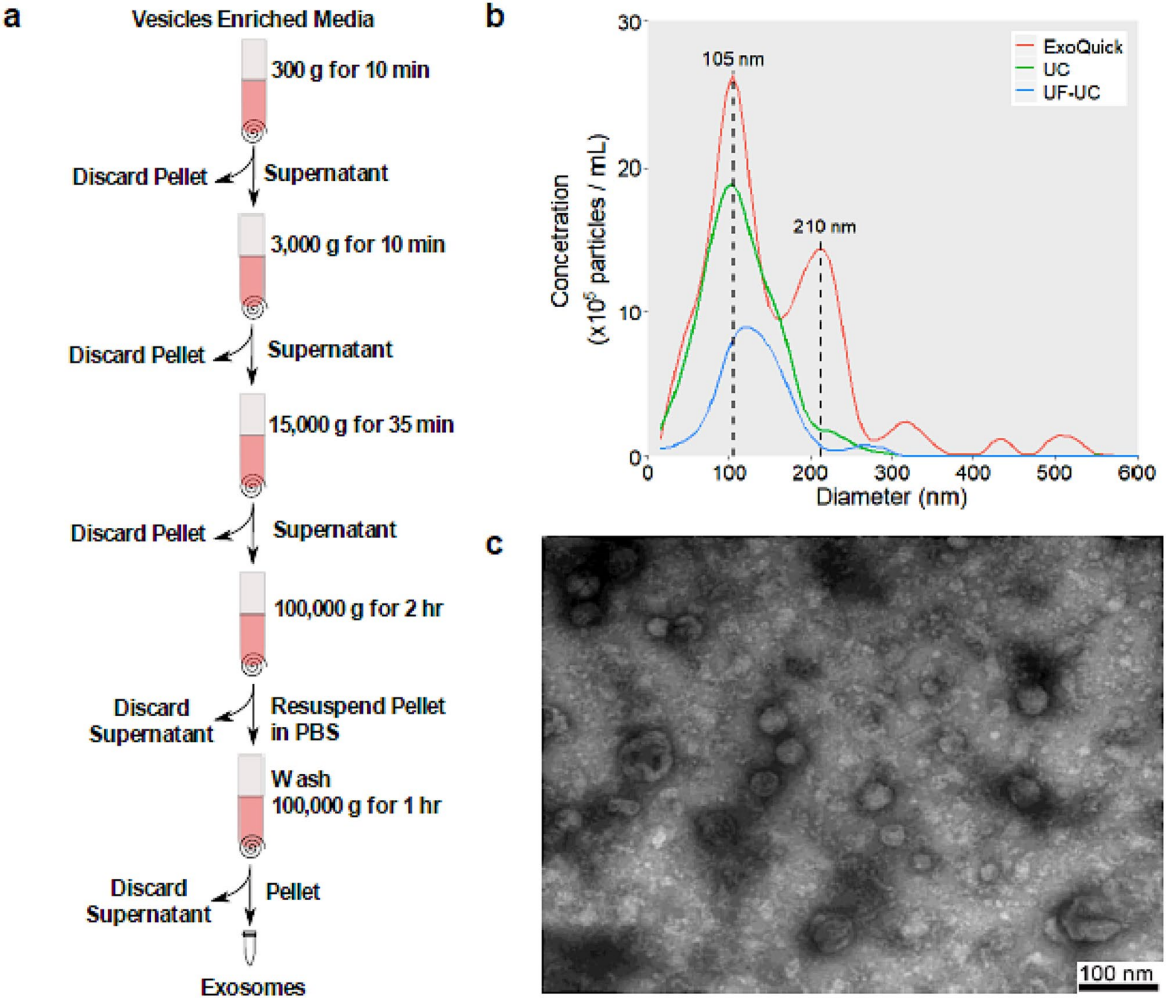
(**a**) Exosome UC isolation protocol. (**b**) NTA analysis of exosomes isolated by ExoQuick (red), UC (green), and UF–UC (blue). (**c**) TEM images of exosomes isolated using the UC method show particles of an average size of 45.5 nm and a size range of 29–86 nm.

Cell culture media was harvested after 48 h of incubation and subject to UC to obtain isolated exosomes (Fig. 1a). 100 mL of cell culture supernatant was collected and immediately centrifuged at 300*g* for 10 min. This was followed by a 3,000*g* spin on a Sigma13190 rotor (MBI) for 10 min, then a 15,000*g* centrifugation for 35 min (SW28 Ti rotor, Beckman Coulter). The supernatant was further centrifuged at 100,000*g* for 2 h (SW28 Ti rotor, Beckman Coulter). Exosomes were pelleted and collected after the 100,000*g* spin. The collected exosomes were washed by centrifuging at 100,000*g* for 1 h (SW55 Ti rotor, Beckman Coulter) and finally resuspended with PBS.

#### ExoQuick

The ExoQuick-TC PLUS kit (Systems Biosciences) was tested for the isolation of exosomes from the MDA cell line. The isolation procedure was done according to the manufacturer’s protocol^23^. In brief, 10 mL of exosome enriched media was centrifuged at 3,000*g* for 15 min. This was followed by the addition of 5 mL of isolation reagent, vortexing for 5 min, and overnight incubation at 4 °C. After 12 h of incubation, the sample was centrifuged at 1,500*g* on a Sigma 13190 rotor for 30 min. The supernatant was discarded, and the pellet was resuspended in 250 μL of resuspension buffer. One unit of washed Microsphere Beads was added to the resuspended sample and placed on a shaker at 450 rpm for 15 min. Finally, exosomes were isolated by spinning the sample at 8,000*g* for 5 min and collecting the pellet.

#### Ultrafiltration–ultracentrifugation (UF–UC)

After centrifuging exosome enriched media at 15,000*g* for 35 min, the supernatant was filtered through a 100 kDa filter (Amicon Ultra-15, Millipore Sigma) by centrifugation at 3,000*g*. The filter concentrated media was then washed by centrifuging at 100,000*g* for 1 h (Figure S1). The pelleted exosomes were resuspended in PBS.

### Quantification of exosomes by nanoparticle tracking analysis (NTA)

The ZetaView nanoparticle tracking microscope PMX-110 (Particle Metrix) was used for determining the concentration and size distribution of exosomes at 85 and 40 camera shutter speeds. Polystyrene beads 102 nm in size (Microtrac 900383) were used to focus the camera and calibrate the instrument.

### Transmission electron microscopy (TEM) of exosomes

Isolated vesicle fractions were fixed in 2.5% glutaraldehyde in 0.1 M sodium cacodylate buffer (pH 7.4). Fixed suspensions containing exosomes were spotted on Formvar^®^ coated copper grids (200 mesh; Canemco, Lakefield, ON, Canada) for 30 s. Samples were negatively stained with 2% uranyl acetate in water for 6 min and dried with filter paper. Vesicles were examined on a transmission electron microscope (JEOL JEM 1230, Japan) operated at 50 kV. The Feret’s diameter of the particles was analyzed using ImageJ software^24^.

### Characterization of exosomal protein markers

Marker-based assessment of exosomes isolated from MDA cells using different methods was performed using the commercially available Exo-Check Exosome Antibody Array kit (System Biosciences, Palo Alto, CA) according to the manufacturer’s protocol. Each array has 12 pre-printed spots and features 8 antibodies for known exosome markers (CD63, CD81, ALIX, FLOT1, ICAM1, EpCAM, ANXA5 and TSG101), a GM130 cis-Golgi marker to monitor any cellular contamination in exosome isolations, a labeled positive control for HRP detection, and a blank spot as a background control.

### In-solution digestion of proteins

Isolated exosomes in 200 μL of PBS were lysed with 50 μL of the solubilization buffer consisting of 8 M urea, 100 mM HEPES, 5% glycerol, and a surfactant (any of 0.5% *n*-dodecyl β-d-maltoside (DDM), 0.5% Digitonin or 0.075% Triton X-100). Samples were then reduced using 4 µL of 100 mM TCEP solution and incubated at 25 °C for 55 min on a shaking plate at 450 rpm. Samples were then alkylated with 4 µL of 500 mM iodoacetamide (in H_2_O) solution and incubated at 25 °C for 55 min on a plate shaking at 450 rpm. Finally, proteins were digested using 1.5 µL of 0.3 µg/µL trypsin/Lys-C solution (Trypsin/Lys-C Mix, Promega V5072) and incubated at room temperature for about 20 h. 2 µL of 100% formic acid was added to samples, which were then vortexed and centrifuged at 10,000*g* for 30 s. Samples were desalted using C18 TopTips (Glygen) columns, as per the manufacturer’s instructions, then vacuum dried.

### Nano-LC–MS/MS

Protein samples (about 5 μg of protein) were analyzed by an Orbitrap Fusion mass spectrometer (Thermo Fisher Scientific) coupled to an UltiMate 3000 nanoRSLC (Dionex, Thermo Fisher Scientific). Peptides were separated on an in-house packed column (Polymicro Technology), 15 cm × 70 μm ID, Luna C18(2), 3 μm, 100 Å (Phenomenex) employing a water/acetonitrile/0.1% formic acid gradient. Samples were loaded onto the column for 105 min at a flow rate of 0.30 μL/min. Peptides were separated using 2% acetonitrile in the first 7 min and then using a linear gradient from 2 to 38% of acetonitrile for 70 min, followed by a gradient from 38 to 98% of acetonitrile for 9 min, then at 98% of acetonitrile for 10 min, followed by a gradient from 98 to 2% of acetonitrile for 3 min and wash 10 min at 2% of acetonitrile. Eluted peptides were directly sprayed into a mass spectrometer using positive electrospray ionization (ESI) at an ion source temperature of 250 °C and an ion spray voltage of 2.1 kV. The Orbitrap Fusion mass spectrometer was run in the top speed mode. Full-scan MS spectra (m/z 350–2000) were acquired at a resolution of 60,000. Precursor ions were filtered according to monoisotopic precursor selection, charge state (+ 2 to + 7), and dynamic exclusion (30 s with a ± 10 ppm window). The automatic gain control settings were 5 × 10^5^ for full FTMS scans and 1 × 10^4^ for MS/MS scans. Fragmentation was performed with collision-induced dissociation (CID) in the linear ion trap. Precursors were isolated using a 2 m/z isolation window and fragmented with a normalized collision energy of 35%.

### Western blotting

About 10 µg of exosomal proteins were lysed with Laemmli sample buffer (Cat. # 1610747) prior to loading onto a 10% TruPAGE™ Precast Gels (Cat. # PCG2001, Sigma-Aldrich). The resolved proteins were transferred to a nitrocellulose membrane (Cat. #1620167, Bio-Rad) using Trans-Blot Turbo Transfer System (Bio-Rad). Membranes were blocked in 1X TBS 1% casein blocker for 2 h at room temperature and incubated with primary antibody (1:1,000 dilution) overnight at 4 °C with slow shaking. The membrane was probed against rabbit anti-ADAM10 antibody (ab1997, Abcam), anti-glypican 1 (GPC-1) antibody (ab199343, Abcam), anti-glucose transporter (GLUT-1) antibody (ab652, Abcam), anti-CD63 antibody (ab216130, Abcam) and CD81 recombinant rabbit monoclonal antibody (SN206-01, ThermoFisher Scientific). Membranes were washed three times in 1 × TBST buffer and incubated with a goat anti-rabbit IgG HPR-conjugated secondary antibody (1:5,000 dilution) for 2 h at room temperature with shaking. Pierce ECL Western Blotting substrate (catalog no. 32209, Thermo Scientific) was used to visualize specific antigen–antibody binding.

### Flow cytometry (FC)

For the validation of protein biomarkers, isolated exosomes were incubated for 40 min with one of the following primary antibodies: Glut-1 (Invitrogen, MA5-31960, SA0377), GLYP-1 (Invitrogen, PA5-86043, polyclonal) and ADAM10 (MyBioSource, MBS435195, polyclonal). The stained exosomes were subsequently incubated in the dark for 40 min with the BV421 conjugated secondary antibody (BD Horizon, 565014, polyclonal), in addition to anti-CD63 (Biolegend, 353008, H5C6) and anti-CD81 antibodies (Biolegend, 349512, 5A6). Data was acquired by a MoFlo Astrios EQ Flow Cytometer (Beckman Coulter). Control samples included an unstained exosome sample and several fluorescence-minus-one samples (Table S1). Flow cytometry data were analyzed using FlowJo analysis software.

### Data processing and statistical analysis

MS raw files were analyzed using the MaxQuant software^25^. Peptides were searched against the human Uniprot FASTA database using the Andromeda search engine^26^, integrated into MaxQuant. Oxidation and N-terminal acetylation were set as variable modifications, while carbamidomethyl was fixed. Trypsin and LysC proteases were chosen as the digestion enzymes with a maximum of 2 missed cleavages. Identified peptides had an initial precursor mass deviation of up to 7 ppm and a fragment mass deviation of 20 ppm. The false discovery rate (FDR) for peptides (minimum of 7 amino acids) and proteins was 1%. A reverse sequence database was used in determining the FDR. For label-free protein quantification, only unique peptides were considered. A contaminant database provided by the Andromeda search engine was used. All proteins matching the reverse database or labeled as contaminants were filtered out. Label-free protein quantification (LFQ) values were obtained through MaxQuant quantitative label-free analysis^25^.

### Bioinformatic analysis

Gene Ontology (GO) functional annotations and Kegg pathway analysis were obtained using David 6.8 database^27^. DisGeNET Curated disease database was used to identify diseases associated with the identified proteins^28^. Information retrieved from previously mentioned databases was visualized using the “ggplot2” R program package^29^. Wilcoxon-Mann–Whitney test was used to assess significance; the test was conducted in the free software R environment^30^.

## Results

### Comparison of exosomes isolation methods

In this study, three different isolation methods (UC, ExoQuick, and UF–UC) were compared for their yield and specificity in capturing exosomes. The ExoCheck kit, NTA, TEM, MS, and FC were utilized to determine the most suitable method for the isolation of exosomes from cell culture media. The ExoCheck kit validated the presence of several external (CD63, EpCAM, ANXA5, CD81, and ICAM) and internal (TSG101, ALIX, and FLOT1) exosomal protein markers in exosomes isolated by three methods (Figure S2). NTA analysis, on the other hand, determined that the highest concentration of vesicles was obtained using the ExoQuick kit (26 × 10^5^ particles/mL) followed by UC (19 × 10^5^ particles/mL) and UF–UC (8 × 10^5^ particles/mL) (Fig. 1b). The average vesicle size of exosomes isolated by the UC method was found to be 105 nm, which is within the accepted 50–120 nm size range of exosomes^1,3^. In contrast, ExoQuick had a significant number of vesicles of size 210 nm, while the average particle size isolated by the UF–UC method was 135 nm. MS analysis of ExoQuick isolated exosomes identified only five proteins, while 986 and 503 proteins were identified using the UC and UF–UC methods, respectively. Out of the top 100 ExoCarta database exosomal proteins, the UC method identified 83 proteins, 40 more than the UF–UC method (Figure S3). Overall, the specificity, high yield, and the number of identified exosomal marker proteins make the UC method the most suitable for exosome isolation. TEM images of UC isolated vesicles confirmed the presence of vesicles with an average size of 45.5 nm that ranged in size from 29 to 86 nm (Fig. 1c).

### Detergent comparison

Three detergents—*n*-Dodecyl β-d-maltoside (DDM), Digitonin, and Triton X-100—were tested for their ability to solubilize exosomal proteins in an in-solution digestion buffer. We observed that DDM was more effective at solubilizing exosomal proteins resulting in the identification of 986 proteins, compared to Triton X-100 and Digitonin, that identified 861 and 778 proteins respectively. DDM also had the highest number of unique proteins (470 proteins) not found by the other detergents (Figure S4). The GO cellular localization analysis of the identified proteins was very similar between the three detergents. The term “Extracellular Exosomes” was the most highly enriched cellular localization term for all three detergents, with DDM having the highest number of proteins under this category (Figure S5).

### Global protein profiling of MCF-10a and MDA-MB-231 exosomes

**Figure 2:**
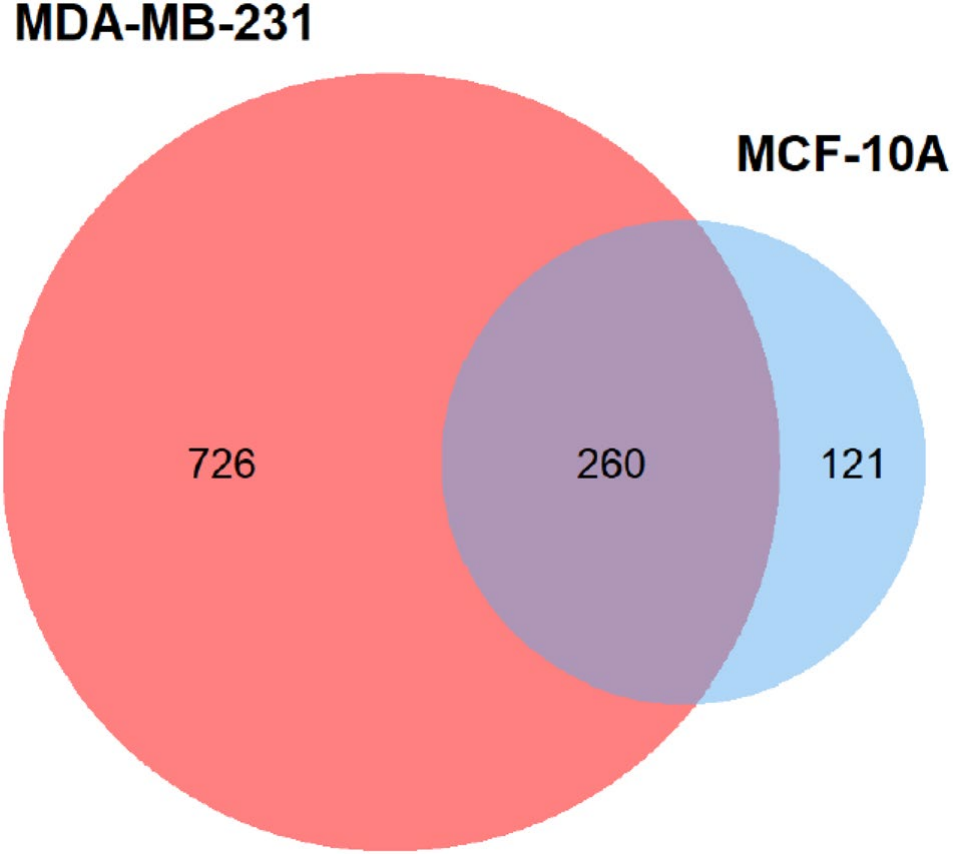
The overlap between MDA-MB-231 and MCF-10A exosomal proteomes. Out of the 986 MDA-MB-231 and the 399 MCF-10A exosomal proteins, 260 proteins were identified in both cell lines.

Each cell line was run in 3 biological replicates. Proteins were considered for analysis if found in at least two biological replicates. MDA and MCF exosomal proteomes were profiled to a depth of 986 and 381 proteins, respectively. There was a significant overlap between the cell lines with 260 exosomal proteins in common (Fig. 2). Out of 726 unique exosomal proteins found only in MDA, 31 were not reported in the exosomal protein database ExoCarta, while MCF had 12 (Tables S2 and S3).

### Functional analysis of identified proteins

Gene Ontology and Kegg Orthology databases were used to annotating the identified proteins. The GO cellular compartment term Extracellular Exosome was the most highly enriched label for both MCF and MDA exosomal proteins (Figure S6). GO terms associated with proteins only found in MCF derived exosomes were mainly implicated in housekeeping and maintenance of basic cellular functions, while annotations of the 726 unique MDA exosomal proteins were associated with two main themes: signaling and motility (Figure S6). GO biological process terms for the MDA cell line related to signaling included: signal transduction, regulation of ERK1 and ERK2 cascade, positive regulation of protein phosphorylation, and proteasome-mediated ubiquitin-dependent protein catabolic process. While MDA cell line signaling pathways identified by Kegg included: ErbB signaling, Proteoglycan, Rap1 signaling, T cell receptor signaling, and PI3K-Akt signaling.

Furthermore, MDA cell line motility associated GO biological process terms include cell adhesion, migration, and establishment of protein localization. Motility related Kegg pathways were associated with endocytosis, adherens junction, focal adhesion, and regulation of actin cytoskeleton.

Interestingly, 36 protein kinases were identified in the MDA exosomal proteome with 19 and 2 being cancer and specifically BC associated, respectively (Table S4). In addition to protein phosphorylation, identified kinases GO biological process terms related to actin remodeling, cell migration, and signaling were among the top 10 terms with the lowest p-values. The Kegg pathway analysis of MDA exosomal kinases included several signaling terms, such as adherence and gap junctions, and NF-kB pathways, in addition to actin regulation and endocytosis motility terms (Figure S7).

### Biomarker selection process

The DisGenet gene-disease database led to the identification of 247 unique MDA exosomal proteins associated with cancer. These cancer-related proteins were found to be significantly enriched in our dataset with a Wilcox p-value of 2.26 × 10^–6^. The protein list was further narrowed by selecting only breast cancer-related ones. The following terms were used as a query in the DisGenet database: "Breast adenocarcinoma," "Invasive Ductal Breast Carcinoma," "Malignant neoplasm of breast," and "Mammary Ductal Carcinoma." These terms reflect the metastatic mammary adenocarcinoma cancer subtype of the MDA cell line^31^. In total, 87 proteins from our dataset were predicted to be relevant to BC disease; these proteins were also found to be highly expressed compared to the rest of the proteome with a Wilcoxon test p-value of 5.5 × 10^–8^ (Figure S8). The GO analysis of the 87 proteins identified 16 metastasis-associated proteins (Table S5), and 62 membrane proteins according to their Biological Process and Cellular localization, respectively. The 62 membrane proteins were further analyzed for the selection of potential biomarkers. The WOLF PSORT protein subcellular localization prediction tool was used to confirm the plasma membrane origin of these proteins. Only proteins with a plasma membrane score of 10 or more were considered. This resulted in 15 predicted plasma membrane proteins (Table 1).

**Table 1:** List of the membrane proteins suggested as potential breast cancer biomarkers.

UniProt ID	Gene	Description
**P02751**	FN1	Fibronectin
**Q9Y2J2**	EPB41L3	Band 4.1-like protein 3
**P02786**	TFRC	Transferrin receptor protein 1
**P35052**	GPC1	Glypican-1
**P21333**	FLNA	Filamin-A
**P11166**	GLUT-1	Glucose transporter 1
**Q9Y6N7**	ROBO1	Roundabout homolog 1
**O14672**	ADAM10	A Disintegrin and metalloproteinase domain-containing protein 10
Q86Y82	STX12	Syntaxin-12
Q16625	OCLN	Occludin
Q9BY67	CADM1	Cell adhesion molecule 1
Q13433	SLC39A6	Zinc transporter ZIP6
Q04721	NOTCH2	Neurogenic locus notch homolog protein 2
P19022	CDH2	Cadherin-2
P33527	ABCC1	Multidrug resistance-associated protein 1

Bolded UniProt IDs are proteins considered for validation due to their relative high abundance.

**Figure 3:**
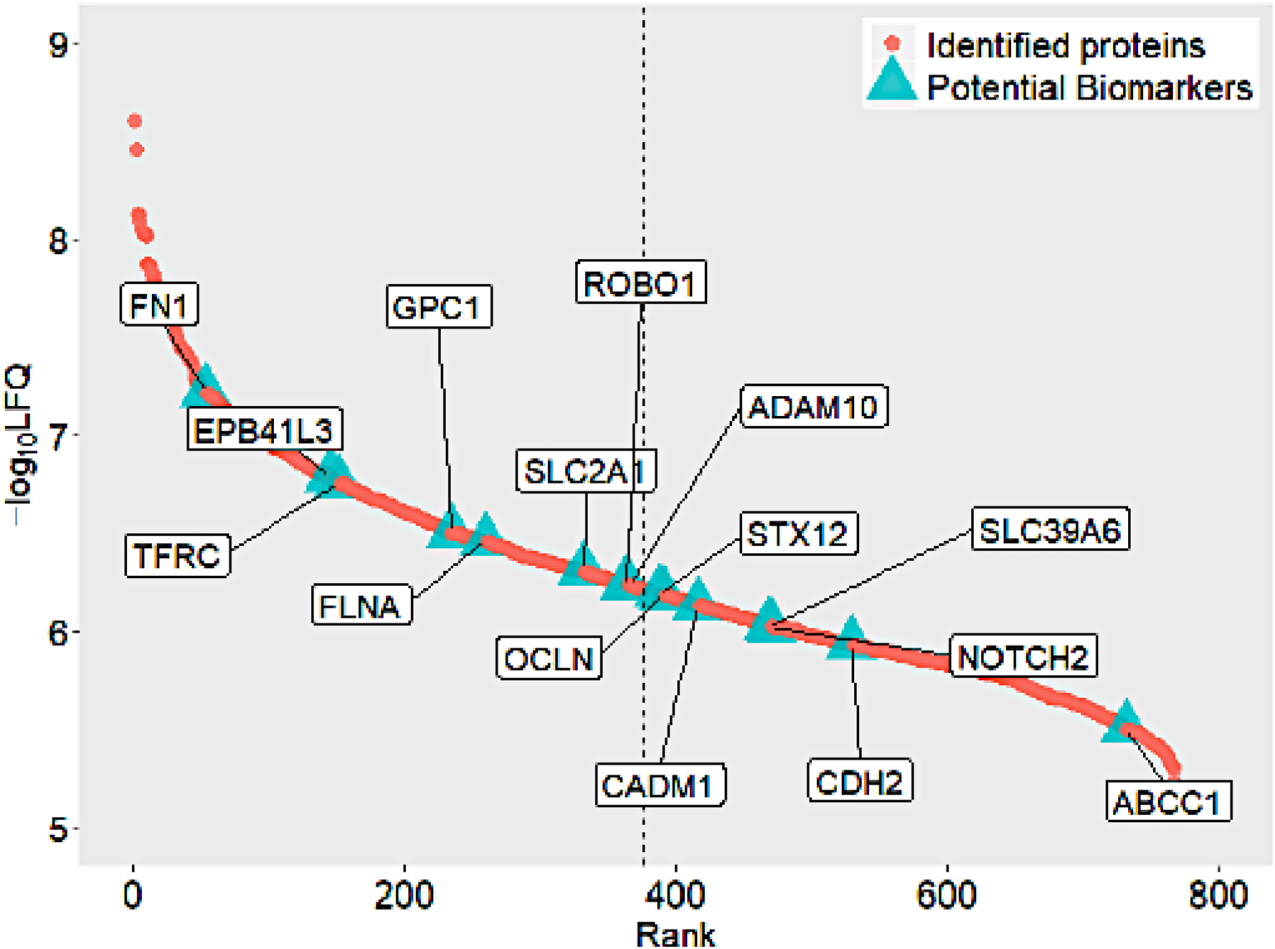
The relative abundance distribution of the 15 potential BC biomarkers over the whole identified proteome. Only abundant proteins, to the left of the dashed lines, were considered for validation.

Furthermore, to ensure the study of easily detectable proteins, confirmed membrane proteins with a − log_10_ LFQ intensities higher than 50% of all identified MDA proteins were selected (Fig. 3, Table 1). The final list of potential BC biomarkers consisted of 8 proteins.

An exhaustive literature search of the eight proteins was performed to better understand their function in BC disease and assess their potential as biomarkers. Glypican 1 (GPC-1), glucose transporter 1 (GLUT-1), also known as solute carrier family 2 (SLC2A1), and a disintegrin and metalloproteinase 10 (ADAM10) were selected for validation as biomarkers. On the other hand, fibronectin (FN1), band 4.1-like protein 3 (EPB41L3), filamin-A (FLNA), roundabout homolog 1 (ROBO1) and transferrin receptor protein 1 (TFRC) did not meet the set criteria described below.

### Western blot analysis of selected exosomal proteins

**Figure 4:**
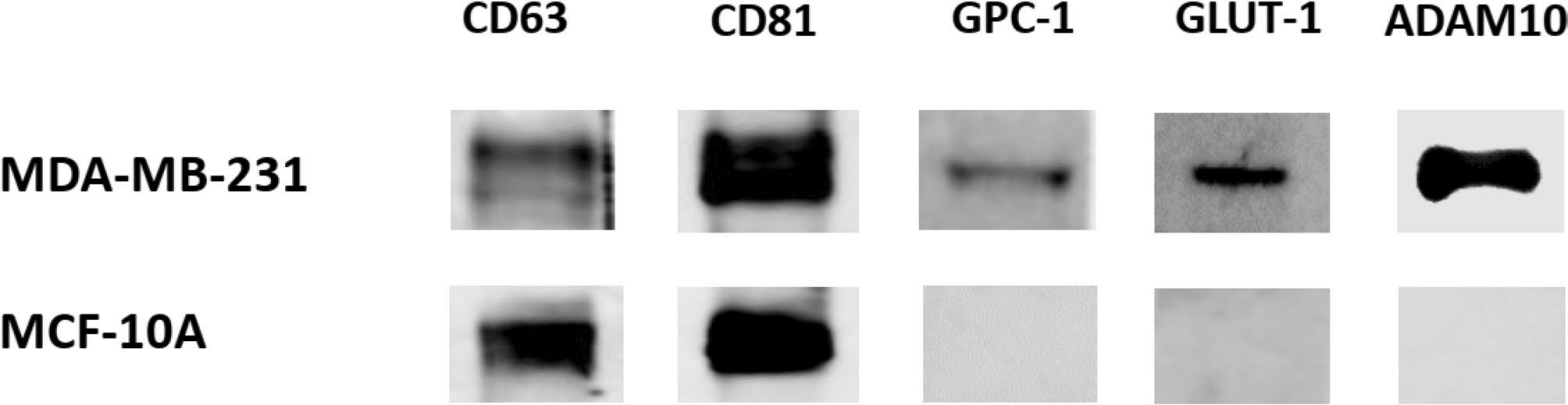
Western blot analysis of CD63, CD81, GPC-1, GLUT-1, and ADAM10 in the exosomes derived from the MDA and MCF cell lines. Full-length gels are shown in Figure S9.

The expression of selected proteins was determined using Western blot analysis. Results revealed the presence of CD63 and CD81 exosomal markers in exosomes derived from both cell lines: MDA and MCF (Fig. 4 and Fig. S9). In contrast, GPC-1, GLUT-1, and ADAM10 were detected only in MDA derived exosomes but were absent in MCF derived sEVs. These results are in agreement with the proteomics data.

### Validation of surface localization of GPC-1, GLUT-1, and ADAM10

**Figure 5:**
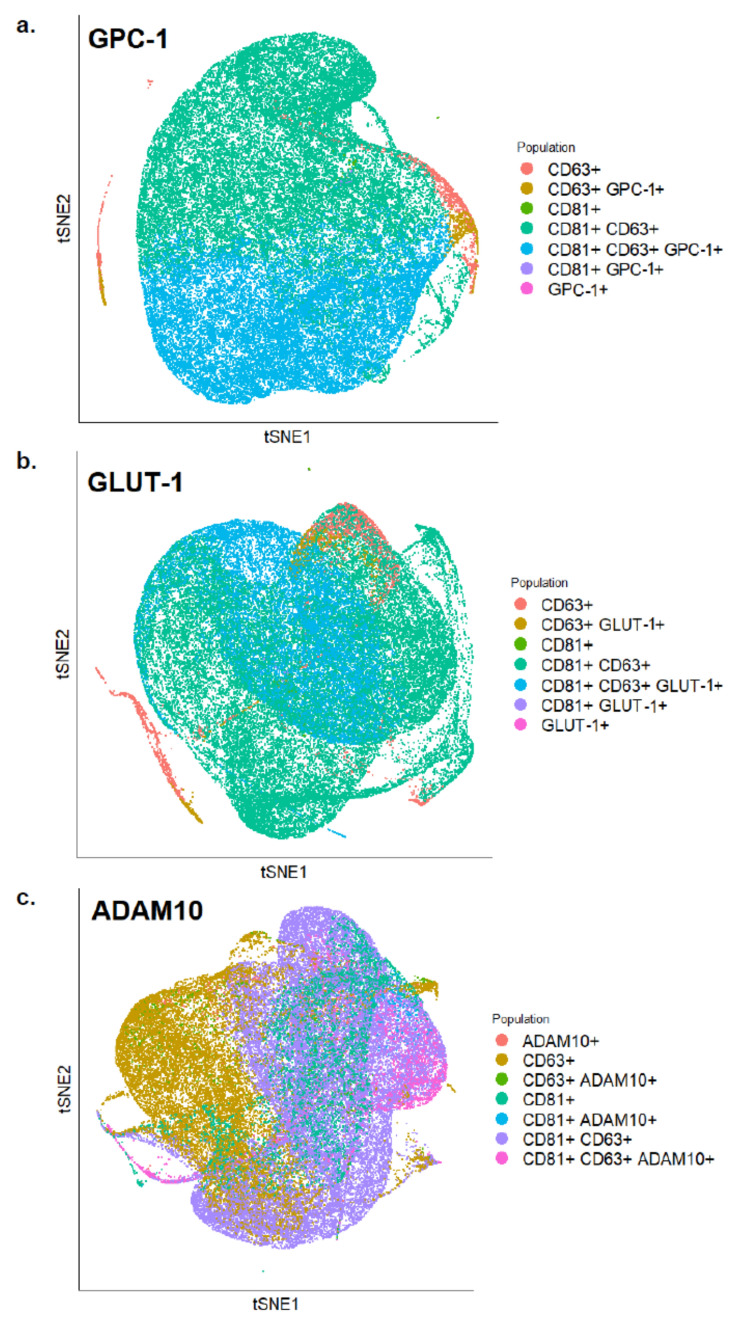
tSNE plots of exosomes analyzed by FACS showing expression levels of (**a**) GPC-1 (**b**) GLUT-1 and (**c**) ADAM10 along with CD81 and CD63 on the surface of BC-derived exosomes.

Flow cytometry analysis was done to further validate the presence of these proteins and determine their relative distribution on exosomes. Flow cytometry experiments were conducted using anti-GPC-1 and anti-GLUT-1 antibodies along with two antibodies against exosomal markers CD63 and CD81. Several fluorescence-minus-one controls were used to ensure proper data analysis (Table S1). The expression of both GLUT-1, GPC-1, and ADAM10 overlapped with CD63 and CD81 positive exosomes (Fig. 5). The exosomal marker CD63 stained 45.8% of vesicles, while 33.6% were positive for CD81. 41% of exosomes were positive for GPC-1, CD63, and CD81. 1.9% of exosomes were positive for CD63 and GPC-1 only. 27.4% of exosomes were stained positive for GLUT-1, CD63, and CD81. 1.86% of GLUT-1 positive exosomes were also positive for CD63 and not CD81%. 5.2% of CD63 and CD81 positive vesicles expressed ADAM10, while 1.5% expressed ADAM10 and CD63. These results confirm the presence of ADAM10, GLUT-1, and GPC-1 on the surface of BC cell-derived exosomes.

## Discussion

In order to analyze the exosomal proteome, we first compared the yield and specificity of different exosomal isolation methods (ExoQuick, UF–UC, and UC) and detergents (DDM, Triton X-100, and Digitonin) for their ability to solubilize exosomal proteins. Although exosomal protein markers were detected in all three exosome isolation methods (Figure S2), the UC technique was ideal for this study due to (1) its specificity in isolating uniform vesicles around 105 nm in size (Fig. 1b) and (2) its compatibility with mass spectrometry proteomic analysis, resulting in the highest number of identified proteins. On the other hand, DDM was the best detergent for the solubilization of exosomal proteins. The DDM detergent recovered the most proteins and had the highest number of proteins under the “extracellular exosome” cellular localization label for both cell lines; hence, all in-solution digestion experiments were carried out using this detergent.

Using UC, 1,107 proteins from MDA and MCF exosomes were identified in this study, of which 726 were unique to MDA. The significant difference in the number of exosomal proteins identified from each cell might be due to the low number of exosomes produced by MCF. MCF has been shown to produce significantly less small-extracellular vesicles and proteins than MDA^32^. MDA exosomal protein annotations could be grouped into two themes: motility and signaling. This is in line with the reported function of cancer exosomes as promoters of cell motility and metastasis^9,33–36^. The 87 identified BC proteins, in the MDA cell line, were rich in cancer-related proteins, including 16 proteins associated with metastasis (Table S5). Stathmin-1 (STMN1: P16949), also known as oncoprotein 18, is one of the metastasis-associated proteins. It is a cytosolic phosphoprotein that regulates microtubule dynamics^37^. Immunohistochemical analysis of BC tissue exhibited an upregulation of Stathmin-1 compared to non-malignant tissue. High expression of Stathmin-1 in BC patients correlated to lower survival rates^38^. Using Stahtmin-1 expression levels and phosphorylation status, BC patients were classified into high and low-risk groups, highlighting its prognostic value^39^. Furthermore, the Peptidyl-prolyl *cis/trans* isomerase NIMA-interacting protein (PIN1: Q13526) also drives BC metastasis. PIN1 mediates the isomerization of phosphorylated pSer/Thr-Pro motifs^40^. Its expression has been found to be higher in metastatic cancers compared to primary ones^41,42^. In BC MDA cell lines, PIN1 was found to increase cancer cell metastasis and invasion capabilities by activating the NOTCH pathway. PIN1 enhanced NOTCH1 tumorigenic activity by potentiating its cleavage by γ-secretase^43^. PIN1 also promoted the epithelial-mesenchymal transition of MCF-7 BC cells by increasing the transcriptional activity of STAT3, signal transducer and activator of transcription 3, which in turn upregulates the TWIST transcription factor^44^. This protein is being explored as a therapeutic target for cancer. Several PIN1 targeted microRNAs (miR296-5p, miR-200c, and miR-370) inhibit cancer progression by decreasing mRNA levels of PIN1^45–47^.

Kinase proteins play essential roles in cancer processes such as immune evasion, cell cycle regulation, and cell proliferation. The extensive role of protein kinase phosphorylation in most cell signal transductions makes them ideal therapeutic targets^48,49^. Out of the two BC associated kinases, the c-Src (SRC: P12931) was of interest (Table S4). The c-Src proto-oncogene tyrosine kinase regulates functions during tumor cell migration, invasion, proliferation, and metastasis^50^. This kinase has been implicated in the late-onset of BC bone metastasis. c-Src is critical for the survival of BC cells in the bone marrow by suppressing apoptosis-related signals and activating the Akt survival-promoting pathway^51^.

For the selection of potential BC biomarkers for detection and prognosis, the focus was on exosomal proteins with upregulated expression in BC that are reported to further increase in expression as the disease progressed. This led us to select GPC-1, ADAM10, and GLUT-1. The cell surface proteoglycan protein GPC-1 has been reported to be strongly expressed in BC tissue compared to healthy ones; its levels were also higher in patients’ tissue with advanced stages of BC^52^. GPC-1 was found expressed in 98.8% of esophageal squamous cell carcinoma patients’ tumor tissue with higher levels associated with poorer prognosis^53^. ADAM10, also known as a disintegrin and metalloproteinase domain-containing protein 10 or CD156c, is a transmembrane protease protein. The knockdown and selective inhibition of this protein in BC MDA cells reduced their migration ability without affecting cell numbers. ADAM10 was also expressed at a higher rate in high-grade tumors compared to low-grade ones; higher levels of this protein correlated with poor outcomes for basal subtypes of BC patients^54^. While in NSCLC, ADAM10 was found to be significantly higher in cancerous tissue, especially in metastatic tissue (P < 0.05), compared to adjacent non-cancerous samples^55^. Finally, GLUT-1 is a solute carrier family 2 protein that facilitates the transport of glucose across the plasma membranes of mammalian cells^56^. GLUT-1 expression correlated with higher grade BC cancer and increased proliferative activity. The absence of this protein significantly increased disease-free survival in BC patients^57^. Extracellular vesicle levels of GLUT-1 protein have been found to double when A431 epidermal carcinoma cells are programmed to transition from epithelial to mesenchymal cells^58^. In gastric cancer, GLUT-1 levels significantly correlated with the invasion level and clinical state of cancer, and its overexpression resulted in enhanced tumor growth in-vivo^59^. These three functionally relevant and abundant exosomal proteins could be good candidates for minimally invasive BC detection.

Other membrane proteins within the eight selected proteins, such as FN1, EPB41L3, FLNA, ROBO1, and TFRC (Table 1), were not chosen for validation in this study. FN1 is a cell surface binding extracellular glycoprotein^60–62^. This protein has already been proposed as a BC biomarker; FN1 levels were elevated in BC patients’ EVs and had better diagnostic accuracy than their levels in plasma^63^. EPB41L3 and FLNA proteins connect the cytoskeleton to transmembrane proteins inside cells; these proteins are oriented towards the cellular lumen and do not have extracellular domains, making their detection difficult^64,65^. ROBO1 protein has not been reported in exosomes before this study according to our knowledge. High expression of this protein in BC cells, in-vitro, is associated with decreased tumorigenesis, while its low expression in invasive ductal carcinoma patients resulted in poor prognosis^66,67^. Finally, TFRC is a membrane glycoprotein that facilitates the cellular uptake of iron from the transferrin plasma glycoprotein via receptor-mediated endocytosis^68^. The TFRC protein has been explored for cancer therapy drug delivery due to its endocytic abilities^69,70^. In BC, the TFRC protein, along with nine other iron-related proteins, has been used to successfully distinguish between cancerous and non-cancerous BC lesions^71^. This protein was found differentially expressed between normal, benign, in-situ, and invasive carcinomas in breast tissues. Its expression is reported to be elevated in invasive and aggressive BCs compared to other phenotypes^72^. Furthermore, the TFRC protein, along with three other proteins, has also been identified as a candidate biomarker for estrogen and progesterone positive (ER+/PR+) invasive ductal BC using pre-clinical samples^73^. TFRC has already been identified on the surface of MDA derived exosomes^74^; hence, it was not selected for validation.

Based on the results of our study, we hypothesize that GPC-1, ADAM10, and GLUT-1 proteins may be novel potential biomarkers for BC detection and prognosis. Future studies should examine the exosomal presence of these proteins in samples from healthy and BC diseased populations. The ideal model system for such a study would be breast tumor-derived exosomes isolated from the blood of BC patients. Blood, however, is a source of exosomes from all tissue types and not only breast tumors. Conducting such a study on exosomes from diverse tissue sources would complicate the selection process and reduce its specificity to BC disease. Therefore, to identify clinically relevant biomarkers, all our selected proteins in Table 1 may be taken into consideration and eventually efficiently validated using clinical blood samples. Other studies have proposed biomarkers for BC^75^. Here, however, our proposed biomarkers are cell surface membrane proteins that can be easily accessible for diagnostic sampling. These membrane proteins could be integrated into effective and simple biomarker diagnostic assays based on monoclonal antibody and aptamer-based methods^76,77^.

## Conclusion

In this study, we demonstrate the richness of the metastatic breast cancer cell line-derived exosomes in relevant proteins capable of driving cancer metastasis. Interestingly, 36 protein kinases were identified in the MDA exosomal proteome, of which 19 and 2 were associated with cancer and breast cancer, respectively. We expect that our provided data will be a valuable resource for further exploration of exosomal proteins in cancerogenesis. We identified GPC-1, ADAM10, and GLUT-1 as potential triple-negative BC biomarkers based on a strict selection criterion. The presence of the three proteins on BC derived exosomes was validated using Western blot and flow cytometry methods. Our findings justify the further study of exosomes for BC biomarker discovery.

## Supplementary information

Supplementary information.
