# Supplementary material for: The proteomic analysis of breast cell line exosomes reveals disease patterns and potential biomarkers
Source: Sci Rep. 2020 Aug 11;10:13572. doi: 10.1038/s41598-020-70393-4 (PMC7419295; doi:10.1038/s41598-020-70393-4)
Supplement: Supplementary file 1 — Supplementary information. [file 41598_2020_70393_MOESM1_ESM.pdf]

The proteomic analysis of breast cell line exosomes reveals disease patterns and potential biomarkers

Yousef Risha[1], Zoran Minic[2], Shahrokh M. Ghobadloo[3], Maxim V. Berezovski[1,2,3] *

[1]Department of Chemistry and Biomolecular Sciences, University of Ottawa, Ottawa, Canada

[2]John L. Holmes Mass Spectrometry Facility, Faculty of Science, University of Ottawa, Ottawa, Canada

[3]Cellular Imaging and Cytometry Facility, Faculty of Science, University of Ottawa, Ottawa, Canada

Supplementary Figures

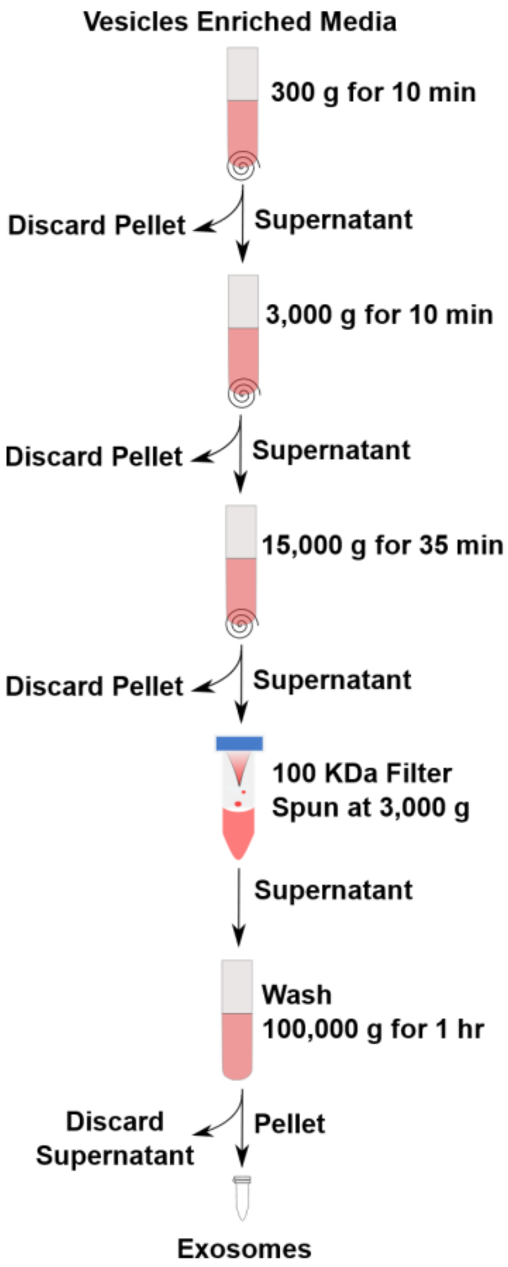

Figure S1. Experimental steps used for the UF-UC exosomes isolation method.

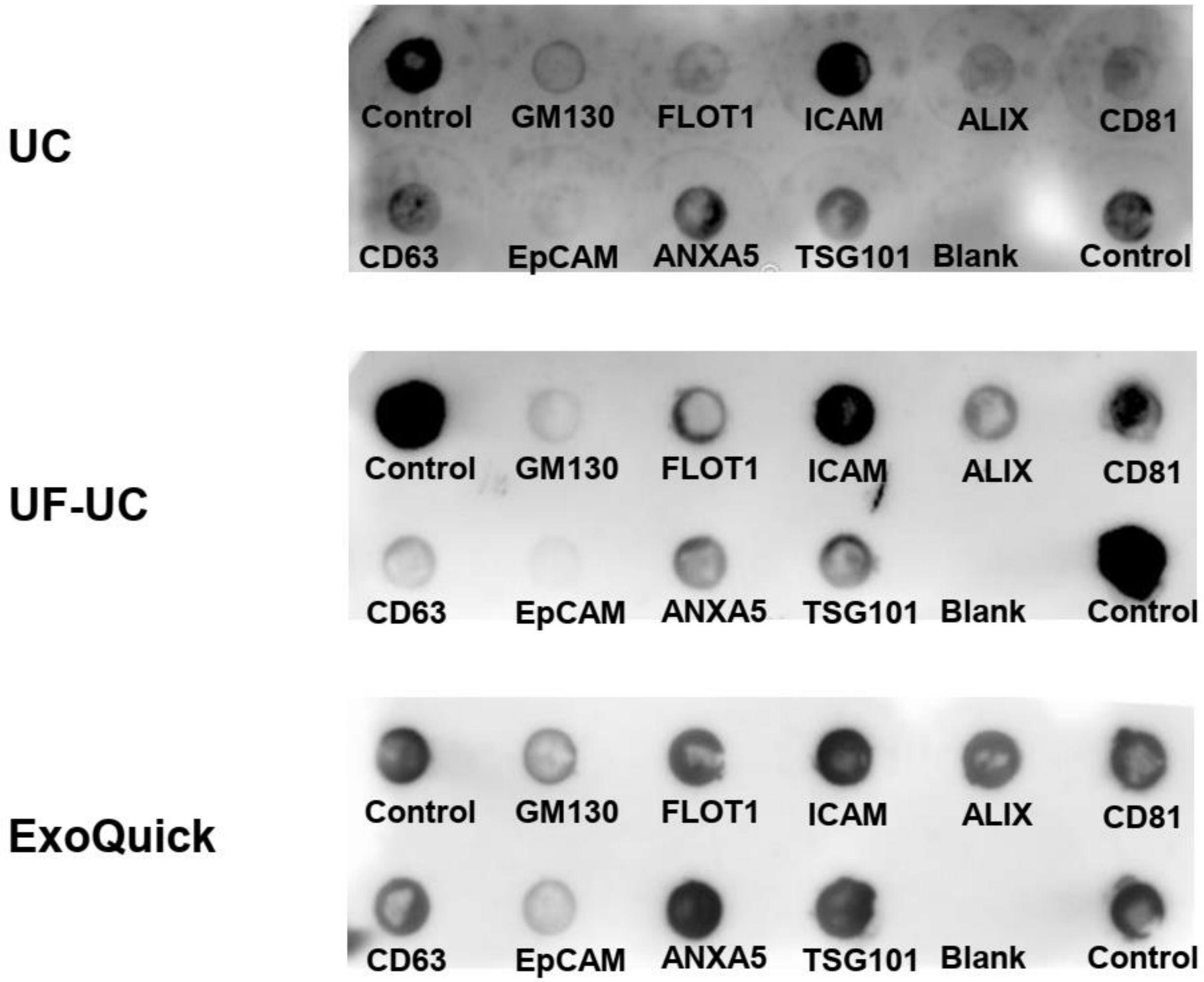

Figure S2. Exo-Check exosome antibody array of five external (CD63, EpCAM, ANXA5, CD81, and ICAM) and three internal (TSG101, ALIX, and FLOT1) exosomal protein markers in exosomes isolated from MDA-MB-231 cells by three methods UC, UF-UC and ExoQuick. GM130 cis-Golgi marker to monitor any cellular contamination in exosome isolations, a labeled positive control for HRP detection, and a blank spot as a background control.

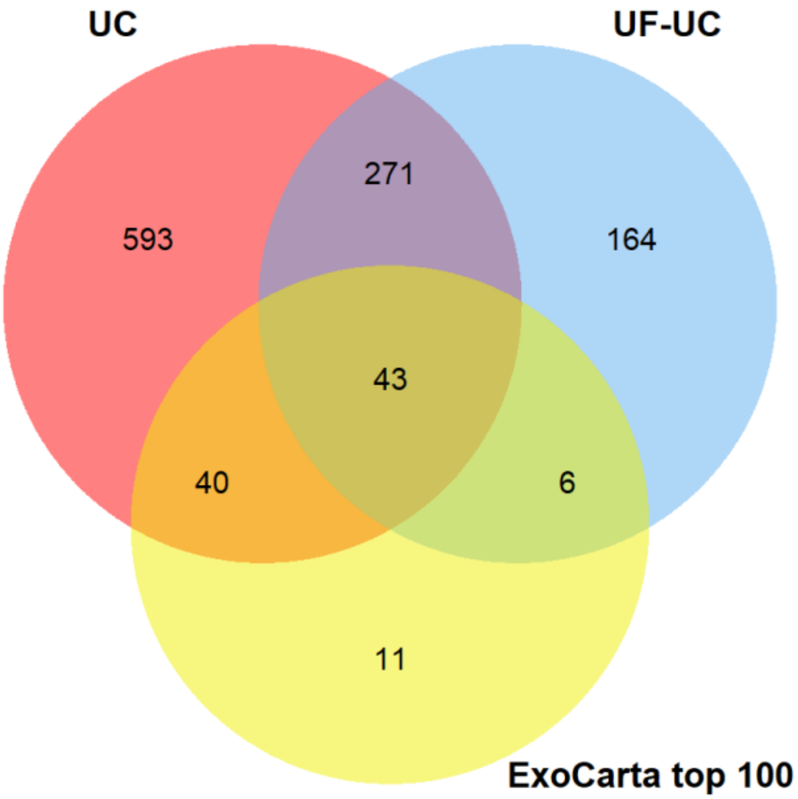

Figure S3. The UC exosome isolation method has more proteins in common with the ExoCarta database Top 100 identified proteins than the UF-UC method.

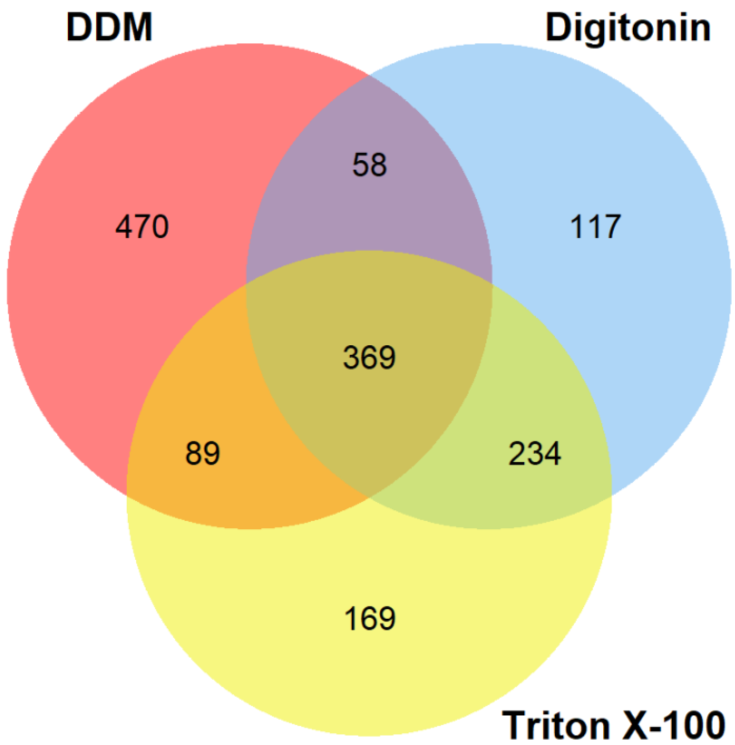

Figure S4. Venn diagram displaying the overlap in exosomal proteins identified using three detergents: DDM, Digitonin and Triton X-100.

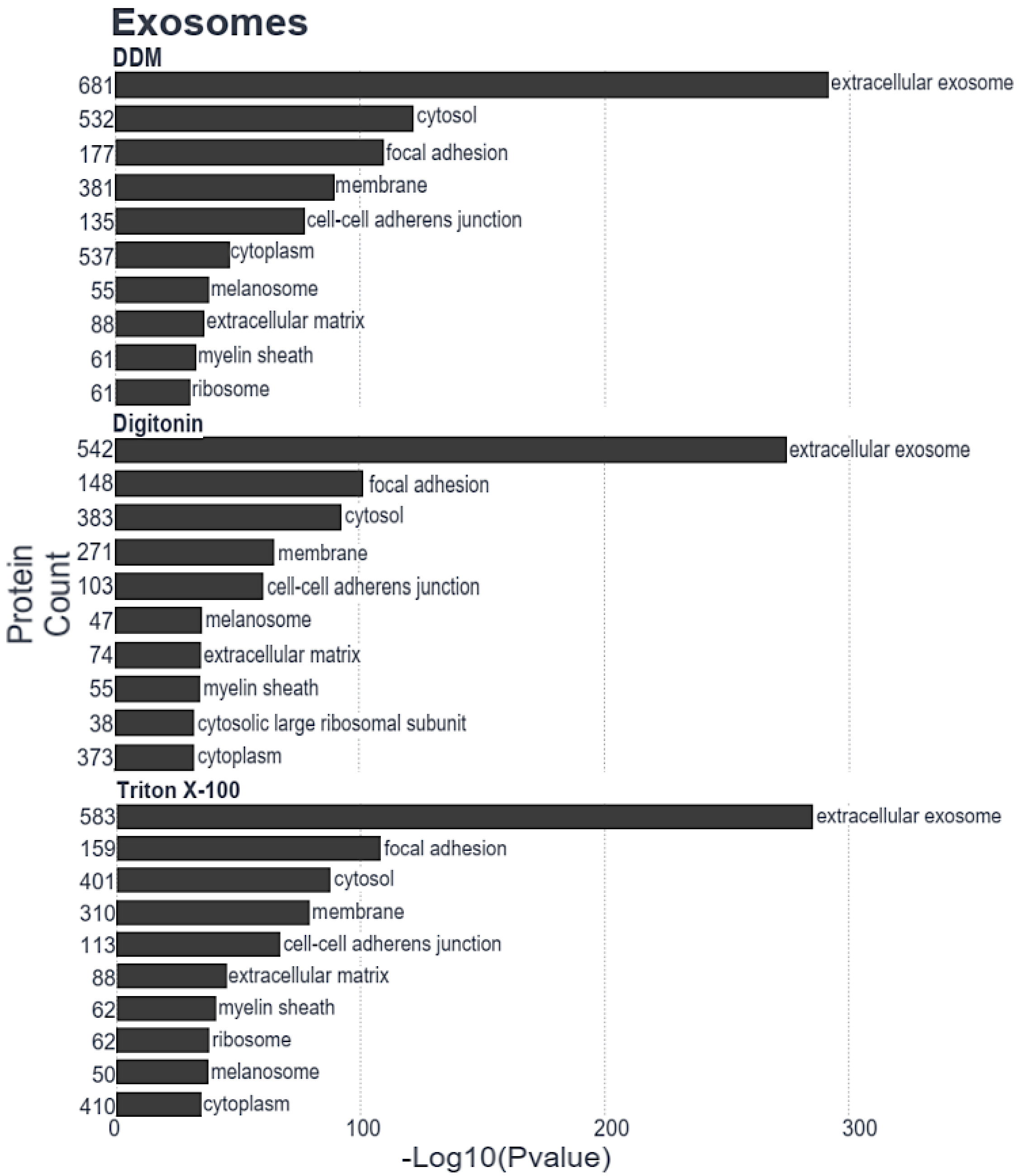

Figure S5. Top 10 GO cellular localization of proteins solubilized by DDM, Digitonin and Triton X-100. P values were calculated using Fisher's exact test and were provided by David's functional annotation tool.

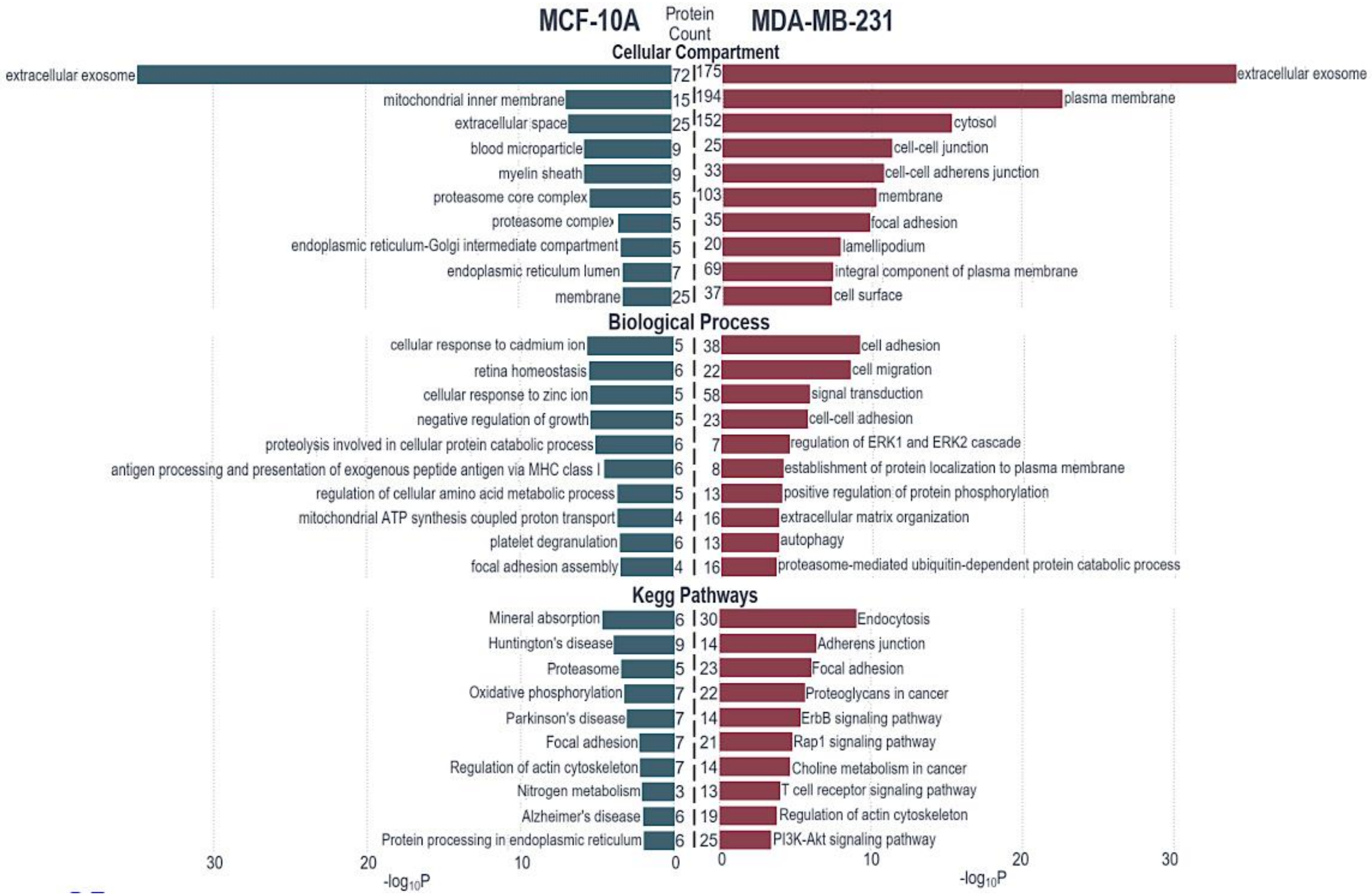

Figure S6. Top 10 GO functional annotation labels, for biological process and cellular compartment, and Kegg pathway analysis for exosome MDA-MB-231 (right) and MCF-10A (left) unique proteins. P values were calculated using Fisher's exact test and were provided by David's functional annotation tool.

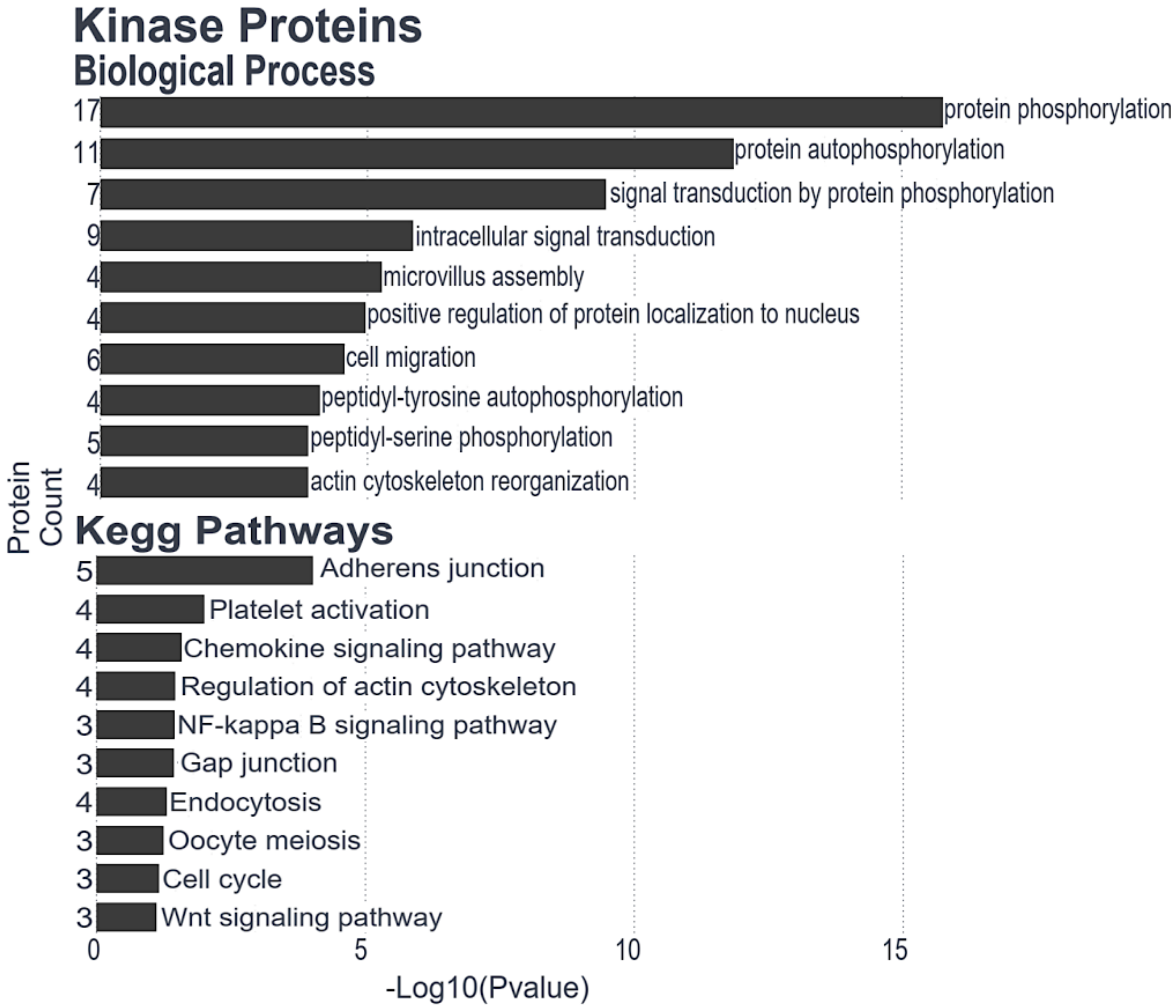

Figure S7. Top 10 GO biological processes and Kegg pathways of identified kinase proteins. P values were calculated using Fisher's exact test and were provided by David's functional annotation tool.

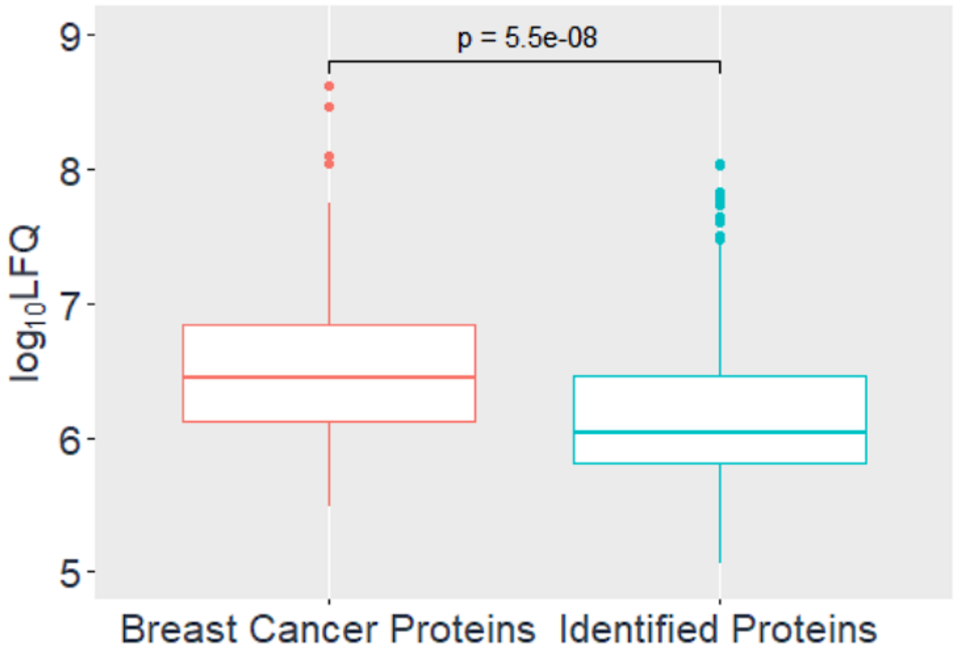

Figure S8. The graph shows the higher expression levels of DisGenet BC identified proteins compared to the rest of the MDA exosomal proteins. The difference was found to be significant according to the Wilcoxon Mann test with a p-value of 0.00028.

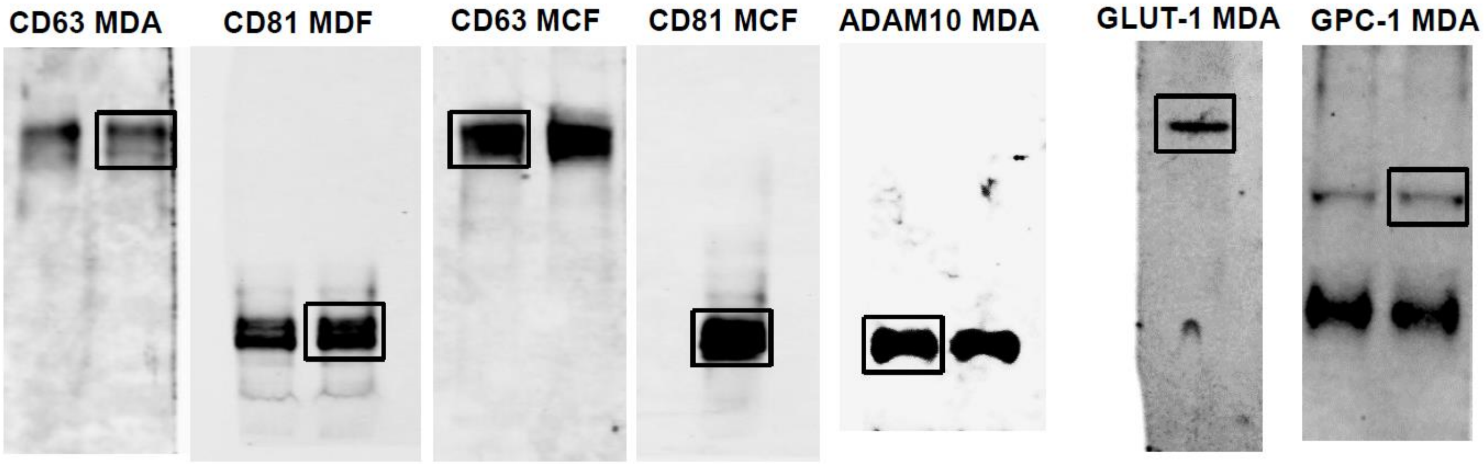

Figure S9. Full-length images of western blot gels. Each sample was run in duplicate except for CD81 MCF and GLUT-1 MDA. All gels were exposed for 30 sec except for ADAM10 MDA, which was exposed for 5 sec. Boxed bands are shown in **Figure 4**.

Supplementary Tables

Table S1. A list of the different control samples used for the validation of GLUT-1 and GLYP-1 using flow cytometry.

Control #	Description
1	Exosomes unstained
2	Exosomes stained with CD81 and GLUT-1
3	Exosomes stained with CD63 and GLUT-1
4	Exosomes stained with CD81 and GLYP-1
5	Exosomes stained with CD63 and GLYP-1
6	Exosomes stained with CD81 and ADAM10
7	Exosomes stained with CD63 and ADAM10
8	Exosomes stained with CD81 and CD63

Table S2: MDA-MB-231 identified exosomal proteins not previously reported in the exosomal protein database ExoCarta.

UniProt ID	Gene	Description
P47813	EIF1AX	Eukaryotic translation initiation factor 1A, X-chromosomal
O60232	ZNRD2	Protein ZNRD2
P0DJJ0	SRGAP2C	SLIT-ROBO Rho GTPase-activating protein 2C
O95218	ZRANB2	Zinc finger Ran-binding domain-containing protein 2
O95235	KIF20A	Kinesin-like protein KIF20A
P07358	C8B	Complement component C8 beta chain
Q32P51	HNRNPA1L2	Heterogeneous nuclear ribonucleoprotein A1-like 2
P42771	CDKN2A	Cyclin-dependent kinase inhibitor 2A
P49711	CTCF	Transcriptional repressor CTCF
P49768	PSEN1	Presenilin-1
Q8NEV1	CSNK2A3	Casein kinase II subunit alpha 3
Q05519	SRSF11	Serine/arginine-rich splicing factor 11
Q14527	HLTF	Helicase-like transcription factor
Q15651	HMGN3	High mobility group nucleosome-binding domain-containing protein 3
Q4VCS5	AMOT	Angiomotin
Q6FI81	CIAPIN1	Anamorsin

Q6P9B6	MEAK7	MTOR-associated protein MEAK7
Q6PKG0	LARP1	La-related protein 1
Q7Z2K8	GPRIN1	G protein-regulated inducer of neurite outgrowth 1
Q8TB73	NDNF	Protein NDNF
Q96GD4	AURKB	Aurora kinase B
Q9BQ16	SPOCK3	Testican-3
Q9H4F8	SMOC1	SPARC-related modular calcium-binding protein 1
Q9H7B2	RPF2	Ribosome production factor 2 homolog
Q9NNW5	WDR6	WD repeat-containing protein 6
Q9NQS7	INCENP	Inner centromere protein
Q9NWQ8	PAG1	Phosphoprotein associated with glycosphingolipid-enriched microdomains 1
Q9NZI8	IGF2BP1	Insulin-like growth factor 2 mRNA-binding protein 1
Q9UET6	FTSJ1	Putative tRNA (cytidine(32)/guanosine(34)-2-O)-methyltransferase
Q9Y383	LUC7L2	Putative RNA-binding protein Luc7-like 2
Q9Y6N7	ROBO1	Roundabout homolog 1

Table S3. MCF-10A identified exosomal proteins not previously reported in the exosomal protein database ExoCarta.

UniProt ID	Gene	Description
P49448	GLUD2	Glutamate dehydrogenase 2, mitochondrial
P04179	SOD2	Superoxide dismutase [Mn], mitochondrial
P0C0L5	C4B	Complement C4-B
P13928	ANXA8	Annexin A8
P14927	UQCRB	Cytochrome b-c1 complex subunit 7
P18859	ATP5PF	ATP synthase-coupling factor 6, mitochondrial
P24539	ATP5PB	ATP synthase F(0) complex subunit B1, mitochondrial
P29034	S100A2	Protein S100-A2
P62328	TMSB4X	Thymosin beta-4
Q15165	PON2	Serum paraoxonase/arylesterase 2
Q5JTV8	TOR1AIP1	Torsin-1A-interacting protein 1
Q9HC84	MUC5B	Mucin-5B

Table S4. Kinase proteins in the BC MDA-MB-231 cell line.

**Unique MDA-MB-231, Kinases**	O94804, O95819, O96013, P06241, P06493, P07947, P07948, P10644, P12277, P12931, P13861, P17612, P19784, P30085, P42771, P43250, P48426, P68400, P78527, Q13308, Q16513, Q7KZI7, Q7L7X3, Q8N4C8, Q96B97, Q96GD4, Q99755, Q99986, Q9H2G2, Q9H8S9, Q9P289, Q9UIG0, Q9UKE5, Q9UKS6, Q9UNF0, Q9Y3F4
**Cancer Kinases**	O95819, P06241, P06493, P10644, P12277, P12931, P14618, P15531, P17612, P19784, P22392, P29966, P48426, P68400, P78527, Q7KZI7, Q96B97, Q96GD4, Q9Y3F4
**BC Kinases**	P12931, P42771

Table S5. List of BC and BC metastasis associated proteins identified using the DisGenet database.

**BC Proteins**	O00299, O00571, O14672, O14745, O43175, O43390, O75340, P02751, P02786, P04075, P04899, P06733, P06744, P07195, P07741, P07900, P08670, P09211, P09874, P0DMV9, P11166, P11387, P11388, P11940, P12931, P13639, P13797, P14174, P14866, P16104, P16403, P16949, P17302, P17987, P19022, P21333, P22087, P23381, P23528, P24534, P26358, P33527, P35052, P35222, P35579, P42771, P43487, P46781, P48643, P49327, P52565, P55010, P61978, P62081, P62241, P62701, P62750, P62753, P62805, P62899, P67809, P84077, Q02952, Q04721, Q07955, Q08431, Q13433, Q13526, Q14112, Q15181, Q16625, Q16643, Q86VP1, Q86Y82, Q8IUE6, Q92597, Q99575, Q99816, Q9BY67, Q9NTK5, Q9UBP0, Q9UHI8, Q9UNF1, Q9Y281, Q9Y2J2, Q9Y4F1, Q9Y6N7
**Metastasis Proteins**	**O14672**, O14745, O75340, **P02751**, P07900, P08670, P12931, P16949, P17302, **P19022**, P23528, P35222, P35579, P52565, Q13526, **Q9Y6N7**

Bolded proteins were among the 15 potential BC biomarkers.
